# Supplementary material for: Deletion of wfs1 Impairs Oligodendrocyte Precursor Cells Dorsal Distribution and Myelination Through the wfs1-hmgcs1 Axis in Zebrafish
Source: Biology (Basel). 2026 Aug 16;15(16):1402. doi: 10.3390/biology15161402 (PMC13510151; doi:10.3390/biology15161402)
Supplement: Supplementary file 1 [file biology-15-01402-s001.zip › File S1-Transcriptome sequencing data and differentially expressed genes (DEGs) in wfs1-- zebrafish at 5 dpf/1.GO/wfs1KOvsWT/DOWN/wfs1KOvsWT.down.GOcc_DAG.pdf]

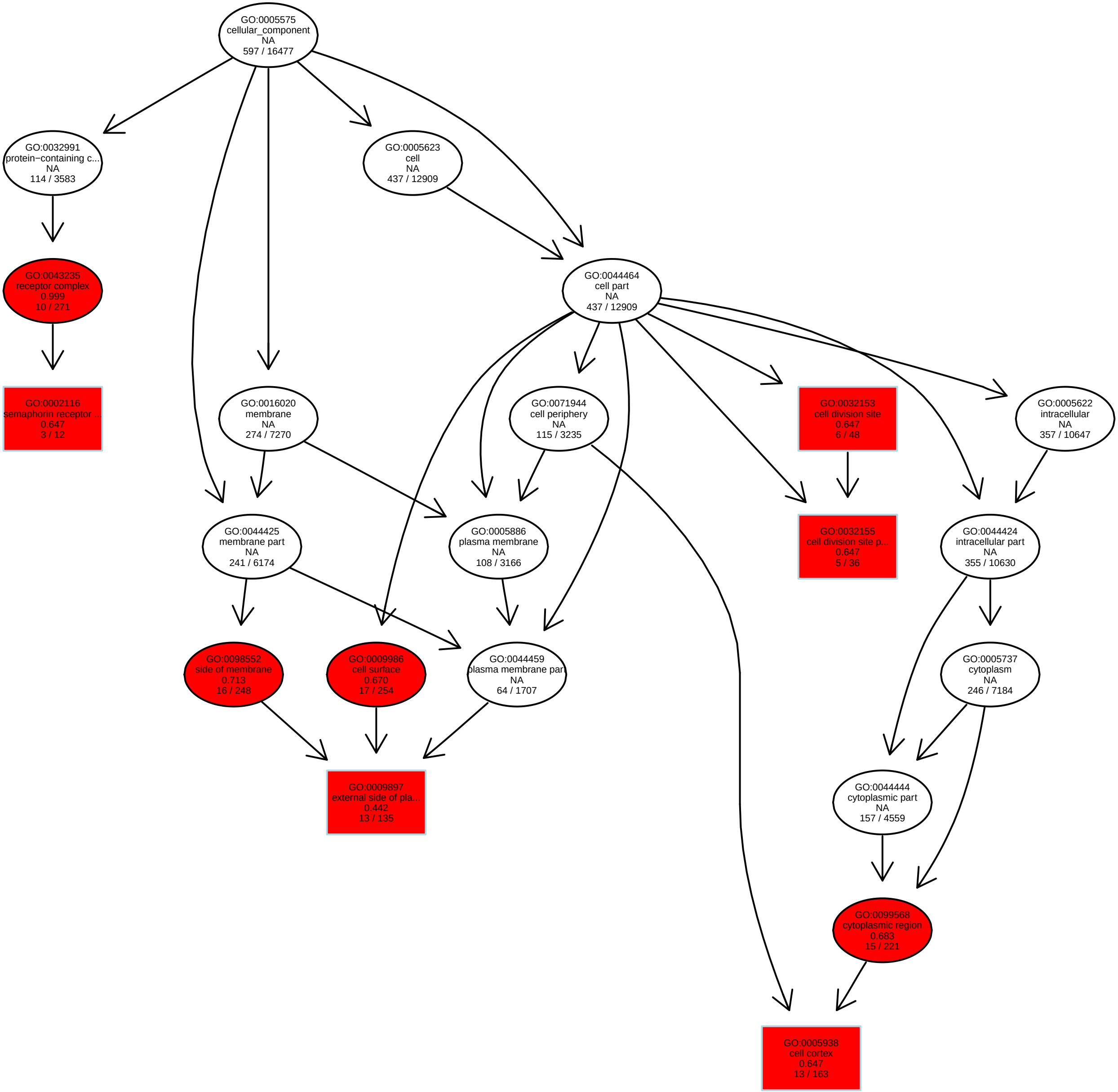
GO:0005575
cellular_component
NA
597 / 16477
GO:0032991
protein−containing c...
NA
114 / 3583
GO:0005623
cell
NA
437 / 12909
GO:0043235
receptor complex
0.999
10 / 271
GO:0044464
cell part
NA
437 / 12909
GO:0002116
semaphorin receptor ...
0.647
3 / 12
GO:0016020
membrane
NA
274 / 7270
GO:0071944
cell periphery
NA
115 / 3235
GO:0032153
cell division site
0.647
6 / 48
GO:0005622
intracellular
NA
357 / 10647
GO:0044425
membrane part
NA
241 / 6174
GO:0005886
plasma membrane
NA
108 / 3166
GO:0032155
cell division site p...
0.647
5 / 36
GO:0044424
intracellular part
NA
355 / 10630
GO:0098552
side of membrane
0.713
16 / 248
GO:0009986
cell surface
0.670
17 / 254
GO:0044459
plasma membrane part
NA
64 / 1707
GO:0005737
cytoplasm
NA
246 / 7184
GO:0009897
external side of pla...
0.442
13 / 135
GO:0044444
cytoplasmic part
NA
157 / 4559
GO:0099568
cytoplasmic region
0.683
15 / 221
GO:0005938
cell cortex
0.647
13 / 163
